# A reverse transcription loop-mediated isothermal amplification assay for quick detection of tomato mosaic virus

**DOI:** 10.1371/journal.pone.0304497

**Published:** 2024-06-13

**Authors:** Phostine M. Kirasi, Elijah M. Ateka, Edith K. Avedi, Hillary K. Yegon, Bramwel W. Wanjala, Hanu R. Pappu

**Affiliations:** 1 Department of Molecular Biology and Biotechnology, Pan African University Institute for Basic Sciences, Technology and Innovation (PAUSTI), Nairobi, Kenya; 2 Department of Phytosanitary and Biosecurity, Kenya Plant Health Inspectorate Service, Nairobi, Kenya; 3 Department of Horticulture and Food Security, Jomo Kenyatta University of Agriculture and Technology, Nairobi, Kenya; 4 Biotechnology Research Institute, Kenya Agricultural and Livestock Research Organization, Nairobi, Kenya; 5 Department of Plant Pathology, Washington State University, Pullman, WA, United States of America; Pan African University of Life and Earth Sciences Institute, PAULESI, NIGERIA

## Abstract

Tomato mosaic virus (ToMV), an economically important virus that affects a wide range of crops, is highly contagious, and its transmission is mediated by mechanical means, and through contaminated seeds or planting materials, making its management challenging. To contain its wide distribution, early and accurate detection of infection is required. A survey was conducted between January and May, 2023 in major tomato growing counties in Kenya, namely, Baringo, Kajiado, Kirinyaga and Laikipia, to establish ToMV disease incidence and to collect samples for optimization of the reverse transcription loop-mediated isothermal amplification assay (RT-LAMP) assay. A RT-LAMP assay, utilizing primers targeting the coat protein, was developed and evaluated for its performance. The method was able to detect ToMV in tomato samples within 4:45 minutes, had a 1,000-fold higher sensitivity than conventional reverse transcription polymerase chain reaction (RT-PCR) method and was specific to ToMV. Furthermore, the practical applicability of the assay was assessed using tomato samples and other solanaecous plants. The assay was able to detect the virus in 14 tomato leaf samples collected from the field, compared to 11 samples detected by RT-PCR, further supporting the greater sensitivity of the assay. To make the assay more amenable for on-site ToMV detection, a quick-extraction method based on alkaline polyethylene glycol buffer was evaluated, which permitted the direct detection of the target virus from crude leaf extracts. Due to its high sensitivity, specificity and rapidity, the RT-LAMP method could be valuable for field surveys and quarantine inspections towards a robust management of ToMV infections.

## 1. Introduction

Plant virus diseases are a serious threat to many cropping systems, resulting in major economic losses [1]. Despite continuous efforts to contain their spread, they remain difficult to control. Additionally, the emergence of novel strains of existing viruses or the discovery of new ones has made continual vigilance important part for preventing their introduction to new areas or regions [2]. Through the international trade of plant materials and seeds, alien pathogens, including viruses, are introduced to new environments, hence the need for enhanced phytosanitary systems [3].

The genus Tobamovirus, family *Virgaviridae*, contains viruses with positive-sense, single-stranded RNA (+ssRNA) [4]. The genus comprises 37 confirmed and several tentative species [4] that are known to infect a wide range of plants, resulting in significant qualitative and quantitative losses. *Tobamoviruses* induce various symptoms in infected plants, and their severity depends on the cultivar, strain of the virus and prevailing environmental conditions [5]. Foliar symptoms of infection include mosaic, mottling and leaf distortion with systemic necrosis and defoliation occurring in cases of severe infection [5]. These viruses spread mechanically through contaminated seeds, planting materials and the use of infected tools [6].

Tomato mosaic virus (ToMV) is one of the most prevalent members of the genus *Tobamovirus* [7]. The virus was first described in the USA in 1909 and has since spread worldwide with substantial yield losses reported in crops of global economic significance [8–12]. ToMV has been identified in tomato and pepper in African countries such as Uganda, Egypt, Algeria, Tunisia, Ethiopia and Nigeria [7, 13–17]. In Kenya, the virus was first detected in tomato collected from the Lake Naivasha area [18], and was estimated to cause up to 91.1% yield loss in susceptible varieties.

Progress has been made in the development of sensitive diagnostic systems for plant viruses [19, 20]. For instance, nucleic acid-based detection methods such a nucleic acid-based hybridization [21], polymerase chain reaction [22], reverse transcription polymerase chain reaction [23], quantitative real-time PCR [24] and next-generation sequencing techniques [25] have revolutionized the diagnosis of plant viruses due to their sensitivity, rapidity and ability to detect multiple viruses simultaneously. However, these methods mostly require advanced laboratory facilities, well-trained staff, and expensive reagents and equipment [26].

Methods for detecting ToMV include serological tests such as enzyme-linked immunosorbent assay (ELISA) [27] and nucleic acid-based techniques such as polymerase chain reaction [28]. Loop-mediated isothermal amplification (LAMP) is a molecular tool that uses a DNA polymerase with strand displacement activity to produce amplicons at a constant temperature [29]. This method is now increasingly being used for virus detection [30] because it has the potential to be less expensive, faster, more robust and more sensitive, with minimal sample processing requirements compared to existing laboratory-based tests [30, 31]. LAMP is therefore adaptable for on-site detection of plant viruses [32, 33]. A variant of the LAMP assay, known as reverse transcription LAMP (RT-LAMP), includes complementary DNA synthesis from the virus RNA template followed by LAMP to identify RNA viruses in plant samples with the aid of a one-step single tube reaction [30]. This technology has been applied to detect more than 100 viruses in 47 genera and, in particular, three Tobamoviruses: tobacco mosaic virus (TMV), tomato brown rugose fruit virus (ToBRFV) and cucumber green mottle mosaic virus (CGMMV) [30]; however, ToMV has not been detected using this tool. This study reports the development of a one-step RT-LAMP assay for the detection of ToMV in host plants. The assay could be used for rapid and sensitive ToMV detection in multiple host plants during surveillance, quarantine inspections and certification programs for virus-free propagating materials.

## 2. Materials and methods

### 2.1. Ethics statement

No permits from government institutions were required to undertake this research. Protected species were not included in this study, nor were the samples obtained from protected areas.

### 2.2. Sources of ToMV

**Fig 1:**
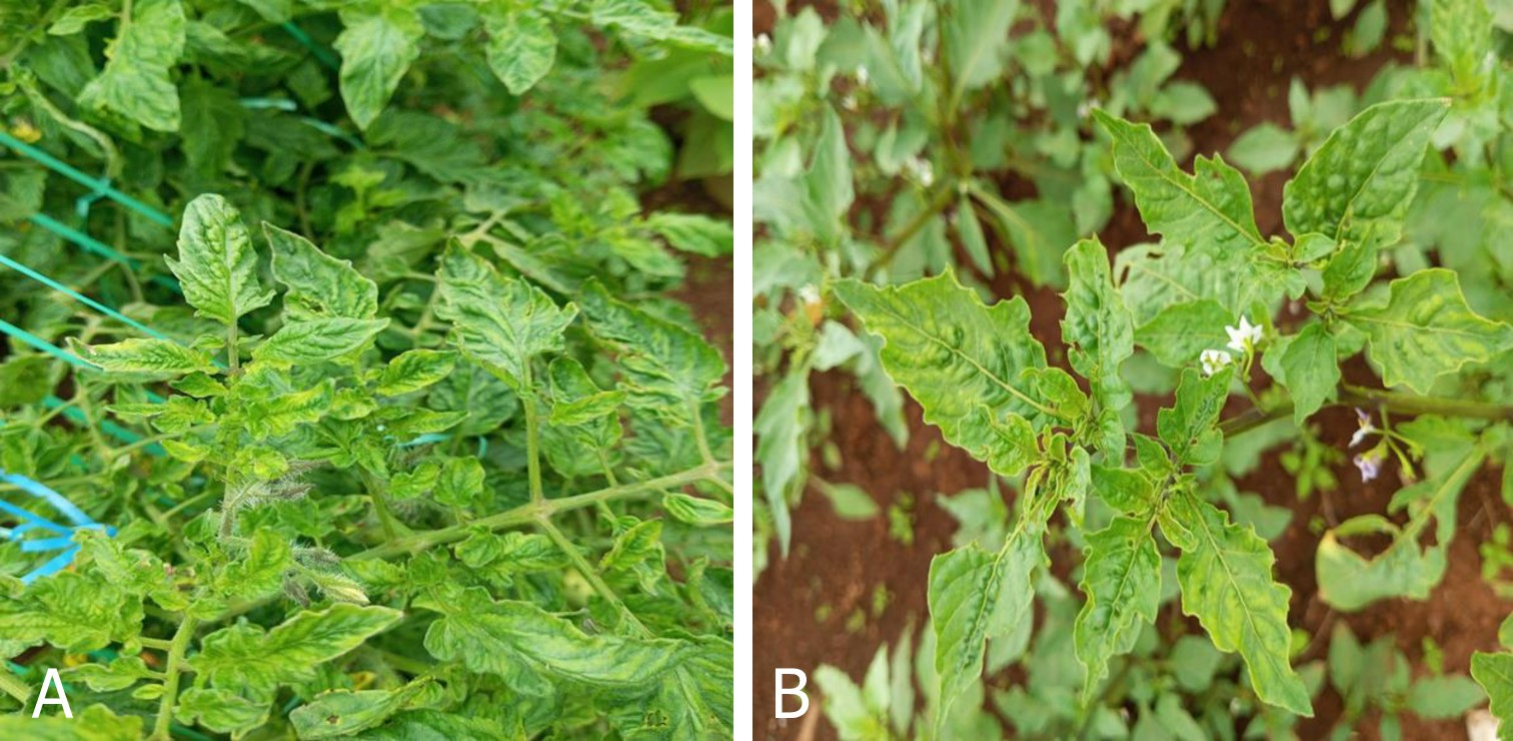
Virus disease symptoms observed in the field. Mosaic, chlorosis, mottling, upward leaf curling, leaf deformation and necrosis symptoms on tomato (A) and black night shade (B).

A field survey was conducted from January to May 2023 in which leaf samples were obtained from 310 tomato (*Solanum lycopersicum*) and 25 black nightshade (*S*. *nigrum*) plants with suspected ToMV infection from four counties in Kenya: Baringo, Kajiado, Kirinyaga and Laikipia. These samples displayed mosaic, mottling, leaf deformation, leaf necrosis, upward curling of leaves and stunting symptoms (Fig 1A and 1B). In each field, representative transects were made, and sampling was performed in each transect in a double diagonal design as described by Sseruwagi *et al*. [34]. The Plants were examined for virus symptoms, and the number of plants exhibiting ToMV symptoms was recorded. Along the transects, five symptomatic plants were sampled, and where no symptoms were observed, asymptomatic plants were collected. Trifoliate leaves from both symptomatic and symptomless plants were sampled, placed in sealable bags, transported in cooler boxes to Kenya Plant Health Inspectorate Service-Plant Quarantine and Biosecurity Station-Muguga (KEPHIS-PQBS) and stored at -80°C prior to laboratory analysis. These samples were tested for ToMV via conventional RT-PCR as reported by Kumar *et al*. [22].

### 2.3. RNA preparation

RNA was extracted from all tomato and black nightshade leaf samples obtained from the field using a modified cetyltrimethylammonium bromide (CTAB) method [35]. The CTAB buffer contained 2% CTAB, 100 mM Tris-HCl, 20 mM EDTA, 1.4 M NaCl, freshly prepared 1% sodium sulphite, and 2% polyvinylpyrrolidone in nuclease-free water (NFW). Briefly, 100 mg of leaf samples were homogenized in 2 μL extraction buffer, transferred in a 1.5 ml micro centrifuge tubes and incubated at 65°C for 15 minutes while mixing at 10-minute intervals. The tubes were centrifuged at 10,000 rpm for 5 minutes, and the supernatants (700 μL) were transferred to new tubes. Equal volume of chloroform:isoamyl alcohol (24:1) were added, mixing was performed by vortexing, and the tubes were thereafter centrifuged at 14,000 rpm for 10 minutes. The aqueous layers were transferred into new tubes, and 700 μL of lithium chloride (stored at 4°C) was added while the tubes were gently inverted. After overnight incubation at 4°C, RNA was pelleted at 14,000 rpm for 30 minutes at 4°C. The supernatants were discarded, and the pellets resuspended in 200 μL of Tris buffer containing 1% SDS. Then, 100 μL of NaCl and 300 μL of ice-cold isopropanol were added, mixed gently, and incubated at -20°C for 20 minutes. The samples were then centrifuged at 14,000 rpm for 10 minutes at 4°C. The salts were removed, and the pellets were washed with 500 μL of 70% (v/v) ethanol and then centrifuged at 10,000 rpm for 4 minutes. The ethanol was removed and the RNA pellets were air-dried at room temperature for 15 minutes. These were dissolved in 50 μL of NFW, and a NanoDrop2000 (Thermo Fisher Scientific, MA, USA) was used to determine the RNA quality and quantity. The integrity of the RNA was checked by 1.5% agarose gel electrophoresis. The extracted RNA was stored at -80°C and used for RT-PCR and RT-LAMP assays.

### 2.4. Complementary DNA synthesis

First-strand complementary DNA (cDNA) synthesis was done using Moloney murine leukemia virus reverse transcription (New England Biolabs, Ipswich, MA, USA) following the manufacturer’s protocol. The first mixture contained 3 μL of RNA, 1 μL of reverse primer (ToMV-R-5’-CGAGAGGGGCAACAAACAT-3’) and 8 μL of NFW to a volume of 12 μL. The mixture was heated at 70°C for 5 minutes and spun down after chilling on ice for 2 minutes. The second master mix was prepared according to NEB’s standard protocol and contained 1μL of dNTPs, 2.5 μL of μMLV- RT buffer, 0.5 μL of μMLV- RT and 4 μL of NFW. The total volume for the cDNA synthesis reaction was 20 μL. The mixture was incubated for 5 minutes at 25°C followed by 60 minutes at 42°C, and the reaction was terminated by heating the mixture at 80°C for 3 minutes. The cDNA was stored at -20°C until use.

### 2.5. Polymerase chain reaction

The 25 μL PCR mixture contained 12.5 μL of 2 × master mix (New England Biolabs, Ipswich, MA, USA), 0.2 μM each forward (ToMV-F-5’-ACCTGTCTCCATCTCTTTGG-3’) and reverse primer (ToMV-R-5’-CGAGAGGGGCAACAAACAT-3), 9.5 μL of NFW and 2 μL of cDNA template. The PCR conditions were as follows: initial denaturation at 94°C for 3 minutes followed by 31 cycles at 94°C denaturation for 30 seconds, annealing at 66°C for 30 seconds, extension at 72°C for 40 seconds and a final extension at 72°C for 10 minutes. All PCRs were carried out in an Eppendorf Master Cycler (Eppendorf, Hamburg, Germany), and the products were resolved on a 1.5% agarose gel stained with GelRed (Biotium, Hayward, CA, USA). Gel images were captured using a C280 gel documentation system (Azure Biosystems, CA, USA).

### 2.6. Screening ToMV-infected tomato samples for other pathogens

To support the inoculation experiments and validation of the established RT-LAMP assay, other pathogens, including cucumber mosaic virus (CMV), tomato spotted wilt virus (TSWV), potato leaf roll virus (PLRV), potato virus Y (PVY), TMV, ToBRFV, tomato mottle mosaic virus (ToMMV) and pospiviroids, were analyzed in tomato leaf extracts that tested positive for ToMV by RT-PCR. PLRV, PVY and PVA were indexed using commercial double-antibody enzyme-linked immunosorbent assay (DAS-ELISA) kits (CIP, Lima, Peru) while the rest were detected by RT-PCR with the primers indicated in Table 1.

**Table 1:** List of primers used for screening of ToMV infected samples for any other pathogen.

Virus/viroid	Primer name	Primer sequence (5′-3′)	Product size (bp)	Reference
CMV	CMV-F	TATGATAAGAAGCTTGTTTCGCG	488	[36]
	CMV-R	GCCGTAAGCTGGATGGACAA		
TSWV	TSWV L1	AATTGCCTTGCAACCAATTC	276	[37]
	TSWV L2	ATCAGTCGAAATGGTCGGCA		
TMV	TMV-F	CGACATCAGCCGATGCAGC	880	[22]
	TMV-R	ACCGTTTTCGAACCGAGACT		
ToMMV	ToMMV-F	AAAAGGGCGGTCTAATTTC	600	[38]
	ToMMV-R	TAATTTCGTCCTTTATTAC		
ToBRFV	ToBRFV-F	AATGTC CATGTTTGTTACGCC	560	[39]
	ToBRFV-R	CGAATGTGATTTAAAACTGTGAAT		
Pospiviroids	Pospi1-FW	GGGATCCCCGGGGAAAC	197	[40]
	Pospi1-RE	AGCTTCAGTTGT(T/A)TCCACCGGGT		

### 2.7. Mechanical inoculation of ToMV onto indicator plants

Leaves from four representative tomato samples, that yielded positive results only for ToMV in RT-PCR, were randomly selected, pooled, sap extracted and mechanically inoculated into six indicator plants, namely, *Nicotiana tabacum cv*. *Samsun*, *Petunia x hybrida*, *Capsicum annuum*, *Calibrachoa parviflora* and *S*. *lycopersicum* (Moneymaker and Asilla F1 varieties), in an insect-proof greenhouse at 25–28°C. The experiment was laid out in a completely randomized designed with three replications. The pregerminated seeds and cuttings were mechanically inoculated at two leaf-stage. This involved grinding the infected leaves (1 g) in 1 ml of 0.01 M phosphate buffer at pH 7.2 before they were applied to young leaves previously dusted with carborundum. Noninoculated plants of each of the test plant species were included as negative controls. The plants were monitored weekly, and symptoms were recorded. Three leaves from the top, middle and bottom of each plant were sampled four weeks post inoculation and stored at -80°C for use in the validation of the RT-LAMP assay.

### 2.8. Design of ToMV-specific LAMP primers

The design of ToMV-RT-LAMP primers was based on the conserved regions of ToMV coat protein-encoding gene (JQ966553.1) retrieved from GenBank that served as a template. Four different sets of primers (F3, B3, FIP and BIP) recognizing six distinct sites on the target genes were designed using Primer Explorer V5 (https://primerexplorer.jp/e/) with the default settings. Moreover, two additional loop primers (LF and LB) were designed to increase the reaction speed. The primer design was based on the primer parameters described by Notomi *et al*. [29]. The primer sets, their sizes, sequences and locations relative to JQ966553.1 are given in Table 2.

**Table 2:** RT-LAMP primers used in the detection of tomato mosaic virus.

Primer name	Length(bp)	Sequence (5’-3’)	Primer positions*
ToMV F3	24	ATATGTCTTACTCAATCACTTCTC	213–237
ToMV B3	21	CCTTATAAACATCGCCAGGAA	403–423
ToMV FIP (F2+F1c)	47	CATCGCAATTTGTGTTTTTGTC-	238–259
		ACTGGTTACCTAACGAATTTGTACA	297–321
ToMV BIP (B2+B1c)	41	ATCTGACGGTGCTCTGAG-	385–402
		CAAACACAGCAAGCAAGAACTAC	324–346
ToMV LF	23	CTATAGGGTCAGCCCATACAGAT	260–282
ToMV LB	20	TGTTCAACAGCAGTTCAGCG	347–366

Key

*F3-Forward outer primer, B3-Backward outer primer, FIP-Forward inner primer, BIP-Backward inner primer, LF-Loop forward primer, LB-Loop backward primer. Primer positions are relative to JQ966553.1 available at GenBank database.

### 2.9. Optimization of the RT-LAMP assay for ToMV

Initial optimization was performed on pure RNA isolated from the four ToMV-infected tomato leaf samples that were used in the inoculation experiments: BAR1, BAR2, KAJ1 and LAK3. The 25 μL RT-LAMP reaction comprised 12.5 μL of isothermal master mix ISO-DR004-RT-150 (Optigene Ltd., UK), 0.2 μM each of forward and reverse outer primers (F3/B3), 1.6 μM each of forward and reverse inner primers (FIP/BIP), 0.4 μM each of forward and backward loop primers (LF/LB), 8.8 μL NFW, 0.5 μL tetra methyl ammonium chloride (TMAC) and 1 μL of RNA template. The constituents of the RT isothermal master mix were: a fast, novel proprietary DNA polymerase (G*sp*SSD2.0), proprietary thermostable inorganic pyrophosphate, optimized reaction buffer, MgSO_4_, dNTPs and a double-stranded DNA-binding dye. The experiment was carried out on a Genie II machine (Optigene Ltd., West Sussex, UK) at 65°C for 30 minutes, followed by a dissociation step from 98°C to 80°C with a decrease of 0.05°C/second. Negative controls, i.e nontemplate (ntc), were included in every run. These reactions were carried out in duplicate, and each experiment was repeated once. In addition, parallel quick extraction was performed for the detection of ToMV. This involved grinding 100 mg of leaf sample in 1 μL of freshly prepared alkaline polyethylene glycol (APEG) buffer (6% w/v PEG−200 in 20 mM NaOH) [41]. The tubes were vortexed briefly and incubated for 5 minutes at room temperature. Crude extracts were diluted with NFW at a ratio of 1:10 and used as templates in RT-LAMP assays. The key parameters considered during optimization included the incubation temperature, reaction time, primer specificity, method of RNA extraction and concentration of the TMAC reagent. To assess specificity and confirm that the correct sequence was amplified, RT-PCR was performed with the RT-LAMP outer primers F3 and B3 which served as the forward and reverse primers, respectively. RT-LAMP amplification was monitored by detecting fluorescence signals over a threshold readout in a rechargeable and portable Genie II machine (Optigene Ltd., West Sussex, UK).

### 2.10. Determination of the specificity and sensitivity of the RT-LAMP assay

The specificity of the RT-LAMP assay was established by evaluating its possible cross- reactivity against seven (7) tomato-infecting viruses: ToBRFV, TMV, TSWV, impatiens necrotic spot virus (INSV), CMV, tomato leaf curl Arusha virus (ToLCArV), PVY and a viroid, potato spindle tuber viroid (PSTVd). An exponential increase in fluorescence readings was used to infer positive reactions whereas flat lines where no amplification reaction occurred signified negative results i.e. samples infected with other viruses and negative controls. The time to positivity (tp) was determined by the peak fluorescence ratio on the amplification rate of the melting curves at a threshold of 0.02. The analytical sensitivity of the developed assay was determined using 10-fold serial dilutions of 100 ng/μL total RNA extracted from ToMV-infected tomato leaf sample (BAR1) by the CTAB and APEG quick extraction methods in NFW down to 1× 10^−9^ ng/μL. Each dilution was used as a template in RT-LAMP and RT-PCR assays (for CTAB-extracted RNA). In all runs, water templates and RNA from virus-free samples were included as negative controls.

### 2.11. Comparison of RT-LAMP results with RT-PCR results

The results of the RT-LAMP assay were subsequently compared with RT-PCR results using RNA extracted from the field-collected and greenhouse-maintained samples tested by RT-LAMP. Nontemplate water controls (ntc) as well as positive samples (total RNA from virus-infected tissues) were included in each run.

## 3. Results

### 3.1. Incidence of ToMV

A total of 78 plants, comprising 68 tomato and 10 black nightshade, tested positive for ToMV according to RT-PCR, as indicated by an expected band size of approximately 318 bp on the gel. However, none of the other pathogens tested in the ToMV-infected tomato samples were detected.

### 3.2. Symptomatology

Varied local and systemic symptoms were recorded on the six indicator plants that were mechanically inoculated with ToMV. Local symptoms were expressed on *N*. *tabacum cv*. *Samsun* and *Petunia x hybrida* (necrotic spots that developed into necrosis on leaves and calyxes), whereas systemic symptoms were observed on *N*. *tabacum cv*. *Samsun* (severe mosaic), *Capsicum annuum* (mosaic and yellowing), *Calibrachoa parviflora* (mosaic), and *S*. *lycopersicum* (mosaic, malformation, narrowing of leaves). Plant death was reported in *Petunia x hybrida*.

As expected, no symptoms were detected in Asilla F1, a ToMV-resistant tomato variety. This observation was consistent for the tomato varieties that were not inoculated and were used as healthy controls in the RT-LAMP assay.

### 3.3. Optimization of RT-LAMP reaction conditions for ToMV detection

**Fig 2:**
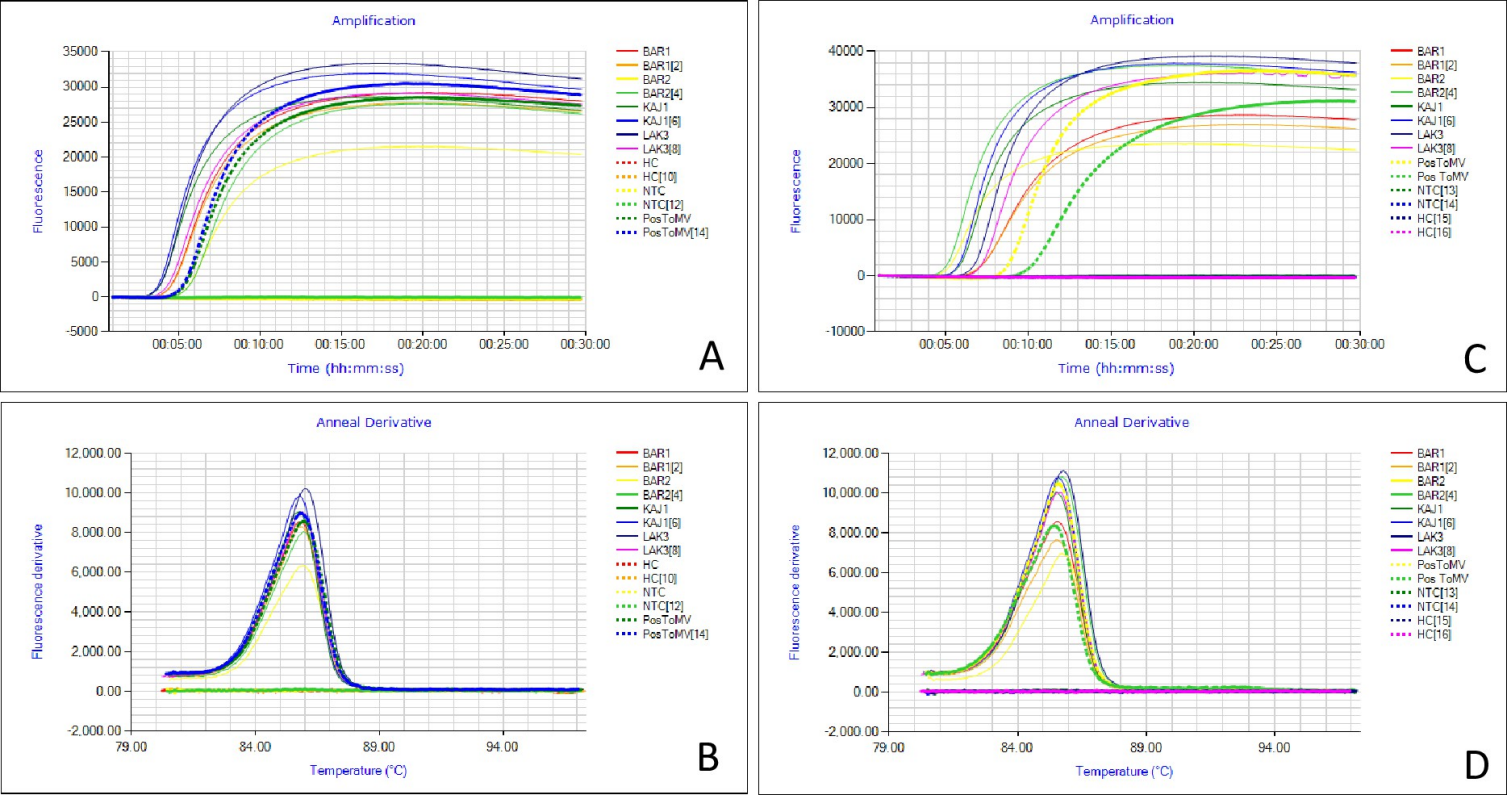
Amplification plot of real-time RT-LAMP for tomato mosaic virus detection. The amplification curves signifies tp values of 4.45–6.30 minutes and 5.00–10.00 minutes for CTAB (A) and APEG-extracted RNA (C), respectively. (B) and (D) Melt curve profiles of RT-ToMV LAMP amplicons. NTC refers to the nontemplate control (water control), HC is the healthy control and, Pos ToMV is the positive control.

The RT-LAMP assay was evaluated for its ability to detect ToMV using a set of six primers designed to amplify highly conserved sequences within the coat protein-encoding gene of ToMV. Several factors were considered during the optimization process to obtain the optimal reaction conditions. The reaction was carried out at 61°C, 63°C, 65°C, 67°C and 69°C for 30 minutes. However, no fluorescence was detected after incubation at 67°C; hence, 65°C was considered the optimal incubation temperature. The tp for the established assay using CTAB-extracted RNA ranged from 4:45 to 6:30 minutes, while the APEG-extracted RNA was amplified in the range of 5:00 to 10:00 minutes (Fig 2A–2C and S1 Table). The melting curve analysis in the Genie II instrument indicated that the RT-LAMP amplification products were specific. The Tm values for the four samples were found to be in the range of 85.8°C to 85.9°C and 85.6°C to 88.77°C for CTAB-extracted and APEG-extracted RNA, respectively, with no annealing derivatives detected in the noninfected samples or the negative controls (Fig 2B–2D and S1 Table). Furthermore, RT-PCR using the outer primers (F3 and B3) confirmed the specificity of the RT-LAMP primers by producing an amplicon size of approximately 200 bp on a 1.5% agarose gel. The addition of 20 mM TMAC to the LAMP master mix was found to be optimal for the assay. This was as a result of the occurrence of nonspecific amplification in nontemplate control reactions that were initially performed without the additive. The addition of TMAC in subsequent analyses eliminated nonspecific amplification.

### 3.4. Sensitivity assay

**Fig 3:**
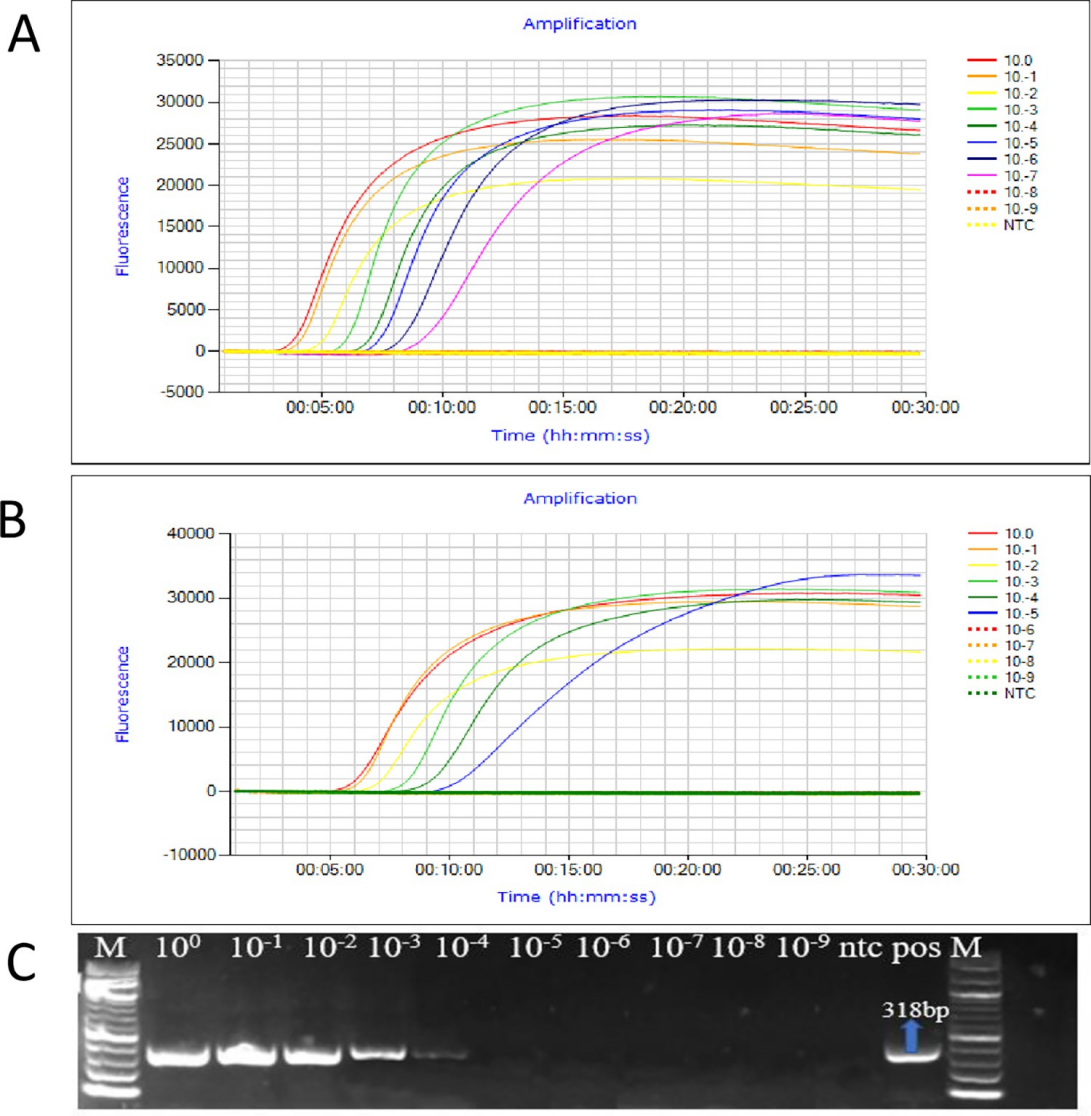
Sensitivity determination of RT-LAMP for ToMV and comparison with RT-PCR. (A) ToMV amplification from purified total RNA extracts by RT-LAMP. Reactions were conducted on RNA extracted from ToMV-infected plant using CTAB and diluted in series from 100 ng/μL to 10^−9^ ng/μL. The RT-LAMP assay was conducted using a Genie II machine (Optigene Ltd., West Sussex, UK). (B) ToMV amplification by RT-LAMP from serially diluted RNA extracted by APEG. (C) ToMV amplification by RT-PCR from purified total RNA; M-1 kb plus DNA ladder (New England Biolabs, Ipswich, MA, USA); ntc-nontemplate control; pos-positive control.

Total RNA extracted from ToMV-infected tomato leaves was serially diluted 10-fold starting from 100 ng/μL to 1× 10^−9^ ng/μL and used to compare the sensitivity of the established RT-LAMP and RT-PCR methods. A pure RNA concentration of up to 1 × 10^−7^ ng/μL could be detected in real time by RT-LAMP (Fig 3A), whereas the limit of detection for RT-PCR was 1× 10^−4^ ng/μL (Fig 3C). Similarly, serially diluted crude RNA extracts in APEG buffer were also tested for ToMV via the established RT-LAMP assay. ToMV was detectable in the RNA samples at dilutions of 1×10^−5^ ng/μL (Fig 3B). Therefore, the RT-LAMP assay was more sensitive than conventional RT-PCR.

### 3.5. Specificity assay

**Fig 4:**
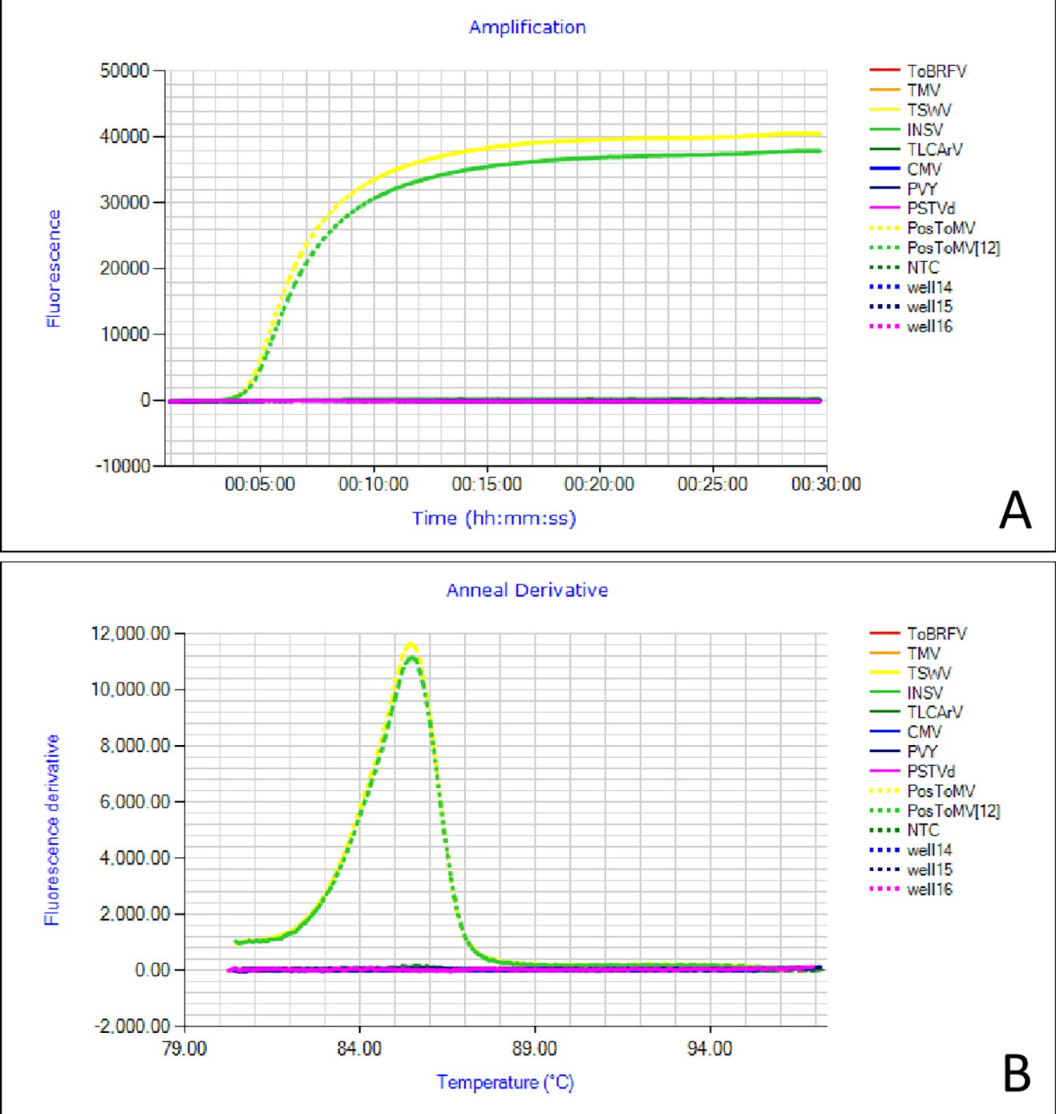
Specificity determination of RT-LAMP for ToMV. (A) Amplification curves and (B) annealing derivatives for tomato mosaic virus. The horizontal lines represent ToBRFV, TMV, TSWV, INSV, ToLCArV, CMV, PSTVd and nontemplate control.

The specificity of the RT-LAMP assay for the detection of ToMV was established by evaluating possible cross-reactivity with selected tomato-infecting pathogens. In ToMV-infected samples, the RT-LAMP product could easily be visualized by increasing fluorescence of the amplification curves, similar to the amplification in positive controls. In contrast, the samples infected with the other viruses and a viroid, namely, ToBRFV, TMV, TSWV, INSV, ToLCArV, CMV, PVY, and PSTVd, did not produce any detectable amplification (Fig 4). This result was consistent with that of nontemplate control reactions. Thus, the RT-LAMP primer set was specific for ToMV.

### 3.6. Evaluating the RT-LAMP assays for field-collected samples

A total of 46 samples comprising 40 newly collected tomato leaf samples and six ToMV-inoculated plants that had been maintained in indicator plants in a greenhouse were used to assess the efficiency of the developed RT-LAMP assay (Table 3). ToMV was detected using APEG-extracted total nucleic acid extracted from leaf samples as previously described. Fourteen (14) field-collected tomato leaf samples and five (5) ToMV-inoculated plants tested positive by RT-LAMP. On the other hand, RT-PCR analysis detected ToMV in eleven (11) tomato leaf samples collected from the field and five (5) from the inoculated samples. A resistant tomato line, Asilla F1 tested negative to ToMV using both tests.

**Table 3:** Comparison of RT-LAMP with the APEG extraction method and RT-PCR for the detection of tomato mosaic virus.

Sample code	Region of sampling	RT-LAMP	RT-PCR	Sample code	Region of sampling	RT-LAMP	RT-PCR
T1	Laikipia	-	-	T25	Baringo	+	-
T2	Laikipia	-	-	T26	Baringo	+	+
T3	Laikipia	+	-	T27	Baringo	+	+
T4	Laikipia	-	-	T28	Baringo	+	+
T5	Laikipia	+	-	T29	Baringo	-	-
T6	Laikipia	+	+	T30	Baringo	-	-
T7	Laikipia	-	-	T31	Baringo	-	-
T8	Laikipia	-	-	T32	Baringo	-	-
T9	Laikipia	-	-	T33	Kajiado	-	-
T10	Laikipia	-	-	T34	Kajiado	-	-
T11	Laikipia	+	+	T35	Kajiado	+	+
T12	Laikipia	-	-	T36	Kajiado	+	+
T13	Laikipia	+	+	T37	Kajiado	-	-
T14	Kirinyaga	-	-	T38	Kajiado	-	-
T15	Kirinyaga	-	-	T39	Kajiado	+	+
T16	Kirinyaga	-	-	T40	Kajiado	-	-
T17	Kirinyaga	-	-	Pet	KEPHIS	+	+
T18	Kirinyaga	-	-	Cal	KEPHIS	+	+
T19	Kirinyaga	+	+	Cap	KEPHIS	+	+
T20	Kirinyaga	-	-	Tob	KEPHIS	+	+
T21	Kirinyaga	-	-	Tom (MM	KEPHIS	+	+
T22	Kirinyaga	-	-	Tom(AF1)	KEPHIS	-	-
T23	Kirinyaga	+	+	NTC	KEPHIS	-	-
T24	Kirinyaga	-	-	POS	KEPHIS	+	+

T1-T40 field-collected tomato leaf samples; Pet-petunia; Cal-Calibrachoa; Cap-Capsicum; Tob-Tobacco, Tom (MM)-Tomato moneymaker; Tom (A. F1)-Tomato Asilla F1; +-Positive;—negative; NTC-no template control; POS-positive control

## 4. Discussion

Outbreaks of virus diseases have indicated the urgency of controlling virus infections for increased food production. Although the identification of causal agents is key for appropriate disease management, it is difficult to achieve this based on visual observation of symptoms only. Many viruses produce similar symptoms, or the symptoms may be due to other environmental factors [22]. Therefore, it is imperative to establish accurate detection methods for each virus. This study evaluated an improved RT-LAMP-based method for the sensitive and specific detection of ToMV. The performance of the assay was evaluated and validated with both artificially and naturally infected solanaecous plants.

The ToMV RT-LAMP assay developed in this study demonstrated very high specificity, with no positive amplification products produced from the samples infected with other pathogens, including TMV, ToBRFV, TLCArV, TSWV, INSV, CMV, PVY and PSTVd. This is similar to other reported LAMP assays [42–44], where specificity was ascertained using both taxonomically related and nonrelated viruses. The assay had a detection limit of 1.0 × 10^−7^ ng/μL for pure RNA extracted from ToMV-infected leaves, which is 1,000 times more sensitive than standard RT-PCR for ToMV. This finding is consistent with previous studies reported by Wani *et al*. [45] and Caruso *et al*. [46] for prunus necrotic ringspot virus and TSWV, respectively. Among the 40 field-collected tomato leaf samples, only 11 tested positive by RT-PCR. In contrast, ToMV was detected in 14 samples using RT-LAMP further suggesting that ToMV RT-LAMP is more sensitive than ToMV RT-PCR.

The detection of RT-LAMP products was achieved using a fluorescence-reading Genie® II machine with FAM detection channels (Optigene Ltd., West Sussex, UK). This approach offers several advantages to alternative detection methods, such as agarose gel electrophoresis, which is associated with a high risk of contamination as a result of the opening of reaction tubes containing LAMP-amplified products [30]. LAMP assays usually produce more amplicons than does conventional PCR [33]. The simple Genie® II instrument (Optigene Ltd., West Sussex, UK) is supplied with a rechargeable battery, which makes it adaptable for onsite ToMV detection and can also be used in low-resource settings, for instance, places with frequent power failures, as reactions can proceed in the absence of electricity.

One of the technical limitations of the LAMP assay is the occurrence of false positive amplification even in the absence of template DNA, which is caused by the inadvertent interlinkages between FIP and BIP, the longest primers that are needed at greater concentrations than other primers [47]. The RT-LAMP assay was initially performed in the absence of TMAC; however, the results obtained with TMAC-containing RT-LAMP were better than those obtained from TMAC-free experiments, as the latter resulted in nonspecific amplification in some nontemplate control reactions. Additives have been shown to improve the performance of isothermal assays, including LAMP and RT-LAMP, by eliminating the nonspecific reactions [48, 49]. This approach is a simpler and less expensive alternative to other solutions, such as the redesign of primers.

To facilitate and shorten the detection process, a quick RNA extraction method based on APEG, as described by Silva *et al*. [32], was evaluated. This permitted the detection of the virus from infected plant materials in less than 30 minutes. The virus nucleic acids for RT-LAMP reactions isolated from plants may contain significant amounts of phenols and other residues. These compounds inhibit the amplification of DNA targets as a result of the interaction of nucleic acids and proteins in the reaction mixture, leading to the degradation of nucleic acids which compromises the quality of the samples, causing false negative results [50]. The obtained RNA was therefore serially diluted using nuclease-free water to minimize the inhibitory effect of putative plant components, which is in agreement with other previously reported LAMP assays [51–53]. The RT-LAMP assay detected ToMV from the crude lysates down to a dilution of 1.0 × 10^−5^ ng/μL, which was higher than that of PCR. These features reduce the time required for template preparation; hence, the method can be adopted for on-site detection of ToMV infection, as previously reported for other viruses [51].

## 5. Conclusion

In conclusion, the ToMV RT-LAMP assay developed in this study is a specific and sensitive method for the rapid detection of ToMV from various host plants. Therefore, this approach could become a valuable diagnostic tool in ToMV disease surveillance programs in tomato plants, ornamentals and other host plants and in phytosanitary certification programs, hence limiting its introduction and spread.

## Supporting information

S1 Fig (PDF)

S2 Fig (PDF)

S3 Fig (PDF)

S4 Fig (PDF)

S1 Table Amplification time and melting temperatures of the four tomato samples used in optimization. (DOCX)

S1 Raw images (PDF)

## References

[1] Elsharkawy MM, El-Okkiah S, Elsadany AY, Bedier MY, Omara RI, Behiry SI, et al. Systemic resistance induction of tomato plants against tomato mosaic virus by microalgae. Egypt journal of biological pest control. 2022;32(1). doi: 10.1094/PDIS-06-20-1349-RE

[2] Salem N, Mansour A, Ciuffo M, Falk BW, Turina M. A new tobamovirus infecting tomato crops in Jordan. Archives of virology. 2016; 161(2):503–506. doi: 10.1007/s00705-015-2677-7 26586328

[3] Prasanna BM, Carvajal-Yepes M, Kumar PL, Kawarazuka N, Liu Y, Mulema AA, et al. Sustainable management of transboundary pests requires holistic and inclusive solutions. Food security. 2022;14:1449–1457. 10.1007/s12571-022-01301-z.

[4] Lefkowitz EJ, Dempsey DM, Hendrickson RC, Orton RJ, Siddell SG, Smith DB. Virus taxonomy: The database of the International Committee on Taxonomy of Viruses (ICTV). Nucleic acids research. 2018;46(D1):D708–717. doi: 10.1093/nar/gkx932 29040670 PMC5753373

[5] Broadbent L. Epidemiology and control of tomato mosaic virus. Annual review of phytopathology. 1975;(14):75–96.

[6] Dombrovsky A., and Smith E. Seed Transmission of Tobamoviruses: Aspects of Global Disease Distribution. In Advances in seed biology; InTech: London, UK, 2017.

[7] Arinaitwe W, Ochwo-Ssemakula M, Mbewe WK, Sseruwagi P, Kyamanywa S, Erbaugh M, et al. Molecular characteristics of tomato mosaic virus infecting tomato in Uganda. African crop science journal. 2018;26(3):433. 10.4314/acsj.v26i3.8.

[8] Chitra TR, Prakash HS, Albrechtsen SE, Shetty HS, Mathur SB. Infection of tomato and bell pepper by ToMV and TMV at different growth stages and establishment of virus in seeds. Journal of plant pathology. 1999;81(2):123–126.

[9] Bae M, Jo Y, Choi H, Tran PT, Kim KH. First report of tomato mosaic virus isolated from tomato and pepper in Vietnam. Journal of plant pathology. 2019;101(1):181. 10.1007/s42161-018-0127-6.

[10] Ullah N, Akhtar KP, Saleem MY, Habib M. Characterization of tomato mosaic virus and search for its resistance in Solanum species. European journal of plant pathology. 2019;155(4):1195–1209. 10.1007/s10658-019-01848-2.

[11] Aghamohammadi V, Rakhshandehroo F, Shams-Bakhsh M, Palukaitis P. Distribution and genetic diversity of tomato mosaic virus isolates in Iran. Journal of plant pathology. 2013;95(2):339–347.

[12] Soler S, Prohens J, López C, Aramburu J, Galipienso L, Nuez F. Viruses Infecting Tomato in València, Spain: Occurrence, Distribution and Effect of Seed Origin. Journal of phytopathology. 2010;158(11–12):797–805. 10.1111/j.1439-0434.2010.01706.x.

[13] Rabie M, Ratti C, Calassanzio M, Aleem EA, Fattouh FA. Phylogeny of Egyptian isolates of Cucumber mosaic virus (CMV) and Tomato mosaic virus (ToMV) infecting Solanum lycopersicum. European Journal of plant pathology. 2017;149(1):219–225. 10.1007/s10658-017-1164-2.

[14] Nechadi S, Benddine F, Moumen A, Kheddam M. Etat des maladies virales de la tomate et stratégie de lutte en Algérie*. EPPO Bulletin. 2002;32(1):21–24. 10.1046/j.1365-2338.2002.d01-21.x.

[15] Ben Moussa A, Makni M, Marrakchi M. Identification of the principal viruses infecting tomato crops in Tunisia. EPPO Bulletin. 2000;30(2):293–296. 10.1111/j.1365-2338.2000.tb00898.x.

[16] Hiskias Y, Lesemann DE, Vetten HJ. Occurrence, distribution and relative importance of viruses infecting hot pepper and tomato in the major growing areas of Ethiopia. Journal of phytopathology. 1999;147(1):5–11. 10.1046/j.1439-0434.1999.147001005.x.

[17] Arogundade O, Balogun OS, Akinyemi SOS, P LK. Surveys of virus diseases on pepper (Capsicum spp.) in South-west Nigeria. African journal of biotechnology. 2015;14(48):3198–3205. doi: 10.5897/AJB2015.14803

[18] Otieno EA. Identification of tomato mosaic strain of Tobacco mosaic virus (TMV) and its effects on yield of tomato (Lycopersicon esculentum) varieties moneymaker and Roma-vf in kenya. J Agric Environ. 1985;3:56–58.

[19] Liu H, Wu K, Wu W, Mi W, Hao X, Wu Y. A multiplex reverse transcription PCR assay for simultaneous detection of six main RNA viruses in tomato plants. Journal of virological methods. 2019;265:53–58. doi: 10.1016/j.jviromet.2018.12.011 . 30576723

[20] Kolade OA, Popoola AR, Igwe DO, Adediji AO. Molecular Diagnostics for Plant Viruses towards Enhanced Delivery of Disease-Free Planting Materials and Germplasm Exchange. Agricultural Biotechnology, Biodiversity and Bioresources Conservation and Utilization. 2022;179–200. 10.1201/9781003178880-11.

[21] Puchades A V., Carpino C, Alfaro-Fernandez A, Font-San-Ambrosio MI, Davino S, Guerri J, et al. Detection of Southern tomato virus by molecular hybridisation. Annals of applied biology. 2017;171(2):172–178. 10.1111/aab.12367.

[22] Kumar S, Udaya Shankar AC, Nayaka SC, Lund OS, Prakash HS. Detection of Tobacco mosaic virus and Tomato mosaic virus in pepper and tomato by multiplex RT-PCR. Letters in appllied microbiology. 2011;53(3):359–363. doi: 10.1111/j.1472-765X.2011.03117.x . 21740446

[23] Rizzo D, Da Lio D, Panattoni A, Salemi C, Cappellini G, Bartolini L, et al. Rapid and Sensitive Detection of Tomato Brown Rugose Fruit Virus in Tomato and Pepper Seeds by Reverse Transcription Loop-Mediated Isothermal Amplification Assays (Real Time and Visual) and Comparison With RT-PCR End-Point and RT-qPCR Methods. Frontiers in microbiology. 2021;12(April). doi: 10.3389/fmicb.2021.640932 33967980 PMC8096992

[24] Tiberini A, Manglli A, Taglienti A, Vučurović A, Brodarič J, Ferretti L, et al. Development and Validation of a One-Step Reverse Transcription Real-Time PCR Assay for Simultaneous Detection and Identification of Tomato Mottle Mosaic Virus and Tomato Brown Rugose Fruit Virus. Plants. 2022;11(4). doi: 10.3390/plants11040489 35214821 PMC8878898

[25] Maree HJ, Fox A, Al Rwahnih M, Boonham N, Candresse T. Application of hts for routine plant virus diagnostics: state of the art and challenges. Frontiers in plant science. 2018;9(August):1–4. doi: 10.3389/fpls.2018.01082 30210506 PMC6119710

[26] Boonham N, Kreuze J, Winter S, van der Vlugt R, Bergervoet J, Tomlinson J, et al. Methods in virus diagnostics: From ELISA to next generation sequencing. Virus Research. 2014;186:20–31. doi: 10.1016/j.virusres.2013.12.007 . 24361981

[27] Mrkvová M, Hančinský R, Grešíková S, Kaňuková Š, Barilla J, Glasa M, et al. Evaluation of New Polyclonal Antibody Developed for Serological Diagnostics of Tomato Mosaic Virus. Viruses. 2022;14(6):1–15. doi: 10.3390/v14061331 35746802 PMC9228224

[28] Sui X, Zheng Y, Li R, Padmanabhan C, Tian T, Deborah GH, et al. Molecular and biological characterization of tomato mottle mosaic virus and development of RT-PCR detection. Plant disease. 2017;101(5):704–711. doi: 10.1094/PDIS-10-16-1504-RE . 30678578

[29] Notomi T, Okayama H, Masubuchi H, Yonekawa T, Watanabe K, Amino N, et al. Loop-mediated isothermal amplification of DNA. Nucleic Acids Res. 2000;28(12):63–70. doi: 10.1093/nar/28.12.e63 10871386 PMC102748

[30] Panno S, Matić S, Tiberini A, Caruso AG, Bella P, Torta L, et al. Loop mediated isothermal amplification: Principles and applications in plant virology. Plants. 2020;9(4):1–28. doi: 10.3390/plants9040461 32268586 PMC7238132

[31] Bhat AI, Aman R, Mahfouz M. Onsite detection of plant viruses using isothermal amplification assays. Plant biotechnology journal. 2022;20(10):1859–1873. doi: 10.1111/pbi.13871 35689490 PMC9491455

[32] Silva G, Oyekanmi J, Nkere CK, Bömer M, Kumar PL, Seal SE. Rapid detection of potyviruses from crude plant extracts. Analytical biochemistry. 2018;546:17–22. doi: 10.1016/j.ab.2018.01.019 29378167 PMC5873530

[33] Ocenar J, Arizala D, Boluk G, Dhakal U, Gunarathne S, Paudel S, et al. Development of a robust, field-deployable loop-mediated isothermal amplification (LAMP) assay for specific detection of potato pathogen Dickeya dianthicola targeting a unique genomic region. PLoS One. 2019;14(6):1–18. doi: 10.1371/journal.pone.0218868 31233546 PMC6590888

[34] Sseruwagi P, Sserubombwe WS, Legg JP, Ndunguru J, Thresh JM. Methods of surveying the incidence and severity of cassava mosaic disease and whitefly vector populations on cassava in Africa: A review. Virus research. 2004;100(1):129–142. doi: 10.1016/j.virusres.2003.12.021 . 15036844

[35] Lodhi M. A., Ye G.-N., Weeden N. F. & Reisch B. A simple and efficient method for DNA extraction from grapevine cultivars and Vitis species. Plant molecular biology Reporter. 1994;12 (1):6–13.

[36] Liu F, Feng L, Chen X, Han Y, Li W, Xu W, et al. Simultaneous Detection of Four Banana Viruses by Multiplex PCR. Journal of phytopathology. 2012;160(11–12):622–627. 10.1111/j.1439-0434.2012.01943.x.

[37] Mumford RA, Barker I, Wood KR. An improved method for the detection of Tospoviruses using the polymerase chain reaction. Journal of virological methods. 1996;57(1):109–115. doi: 10.1016/0166-0934(95)01975-8 . 8919828

[38] Turina M, Geraats BPJ, Ciuffo M. First report of Tomato mottle mosaic virus in tomato crops in Israel. New disease reports. 2016;33(1):1–1. 10.5197/j.2044-0588.2016.033.001.

[39] Alkowni R, Alabdallah O, Fadda Z. Molecular identification of tomato brown rugose fruit virus in tomato in Palestine. Journal of plant pathology. 2019;101(3):719–723. 10.1007/s42161-019-00240-7.

[40] Verhoeven JTJ, Jansen CCC, Willemen TM, Kox LFF, Owens RA, Roenhorst JW. Natural infections of tomato by Citrus exocortis viroid, Columnea latent viroid, Potato spindle tuber viroid and Tomato chlorotic dwarf viroid. European journal of plant pathology. 2004;110(8):823–831. 10.1007/s10658-004-2493-5.

[41] Hwang H, Bae S-C, Lee S, Lee Y-H, Chang A. A Rapid and Simple Genotyping Method for Various Plants by Direct-PCR. Plant breeding and biotechnology. 2013;1(3):290–297. 10.9787/PBB.2013.1.3.290.

[42] Zhao LM, Li G, Gao Y, Zhu YR, Liu J, Zhu XP. Reverse transcription loop-mediated isothermal amplification assay for detecting tomato chlorosis virus. Journal of virological methods. 2015;213:93–97. doi: 10.1016/j.jviromet.2014.11.013 . 25486081

[43] Suzuki R, Fukuta S, Matsumoto Y, Hasegawa T, Kojima H, Hotta M, et al. Development of reverse transcription loop-mediated isothermal amplification assay as a simple detection method of Chrysanthemum stem necrosis virus in chrysanthemum and tomato. Journal of virological methods. 2016;236:29–34. doi: 10.1016/j.jviromet.2016.07.005 . 27400833

[44] Budziszewska M, Wieczorek P, Obrępalska-Stęplowska A. One-step reverse transcription loop-mediated isothermal amplification (RT-LAMP) for detection of tomato torrado virus. Archives of virology. 2016;161(5):1359–1364. doi: 10.1007/s00705-016-2774-2 . 26887971 PMC4839060

[45] Wani LA, Jawa P, Khan JA. Development of one step colorimetric RT-LAMP assays for rapid detection of Apple mosaic virus and Prunus necrotic ringspot virus. Journal of virological methods. 2023;316(114729,):ISSN 0166-0934. doi: 10.1016/j.jviromet.2023.114729 . 37031745

[46] Caruso AG, Ragona A, Agrò G, Bertacca S, Yahyaoui E, Galipienso L, et al. Rapid detection of tomato spotted wilt virus by real-time RT-LAMP and in-field application. Journal of plant pathology. 2024. 10.1007/s42161-024-01613-3.

[47] Salazar A, Ochoa-Corona FM, Olson JD, Babu B, Paret M. Probing loop-mediated isothermal amplification (LAMP) targeting two gene-fragments of rose rosette virus. PLoS One. 2021;16(11):1–22. doi: 10.1371/journal.pone.0256510 . 34843487 PMC8629277

[48] Ghaith DM, Abu Ghazaleh R. Carboxamide and N-alkylcarboxamide additives can greatly reduce non specific amplification in Loop-Mediated Isothermal Amplification for Foot-and-Mouth disease Virus (FMDV) using Bst 3.0 polymerase. Journal of virological methods. 2021;298(September 2021). doi: 10.1016/j.jviromet.2021.114284 . 34520810

[49] Jang MJ, Kim S. Inhibition of Non-specific Amplification in Loop-Mediated Isothermal Amplification via Tetramethylammonium Chloride. Biochip journal. 2022;16(3):326–333. doi: 10.1007/s13206-022-00070-3 35909465 PMC9326409

[50] Salzman RA, Fujita T, Zhu-Salzman K, Hasegawa PM, Bressan RA. An Improved RNA Isolation Method for Plant Tissues Containing High Levels of Phenolic Compounds or Carbohydrates. Plant molecular biology reporter. 1999;17(1):11–17. 10.1023/A:1007520314478.

[51] Wanjala BW, Ateka EM, Miano DW, Fuentes S, Perez A, Low JW, et al. Loop-Mediated Isothermal Amplification assays for on-site detection of the main sweetpotato infecting viruses. Journal of virological methods. 2021;298(September):114301. doi: 10.1016/j.jviromet.2021.114301 . 34560111 PMC8543070

[52] Tangkanchanapas P, Höfte M, De Jonghe K. Reverse transcription loop-mediated isothermal amplification (RT-LAMP) designed for fast and sensitive on-site detection of Pepper chat fruit viroid (PCFVd). Journal of virolological methods. 2018;259:81–91. doi: 10.1016/j.jviromet.2018.06.003 . 29894712

[53] De Jonghe K, De Roo I, Maes M. Fast and sensitive on-site isothermal assay (LAMP) for diagnosis and detection of three fruit tree phytoplasmas. European journal of plant pathology. 2017; 147(4):749–759.

