# Supplementary material for: A reverse transcription loop-mediated isothermal amplification assay for quick detection of tomato mosaic virus
Source: PLoS One. 2024 Jun 13;19(6):e0304497. doi: 10.1371/journal.pone.0304497 (PMC11175515; doi:10.1371/journal.pone.0304497)
Supplement: S1 Fig — (PDF) [file pone.0304497.s001.pdf]

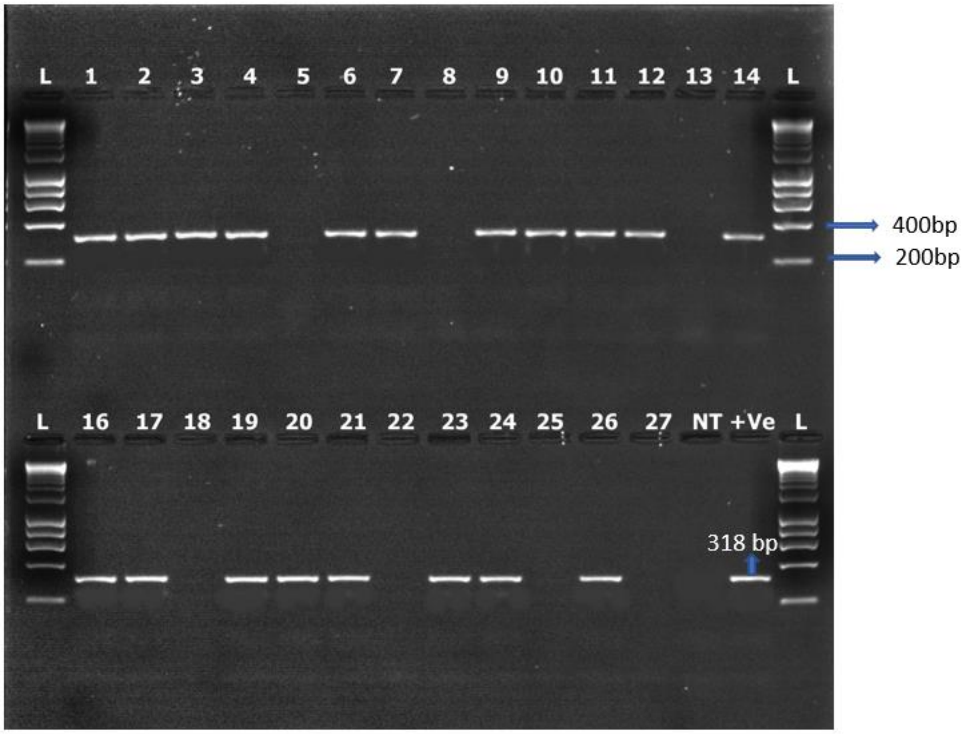


**S1 Fig. Detection of ToMV by RT-PCR.**

L: 1Kb Hyper Ladder (Bioline); lane 1-27 samples; lane 28 –Non-template control; 29-Positive control
