# Supplementary material for: A reverse transcription loop-mediated isothermal amplification assay for quick detection of tomato mosaic virus
Source: PLoS One. 2024 Jun 13;19(6):e0304497. doi: 10.1371/journal.pone.0304497 (PMC11175515; doi:10.1371/journal.pone.0304497)
Supplement: S2 Fig — (PDF) [file pone.0304497.s002.pdf]

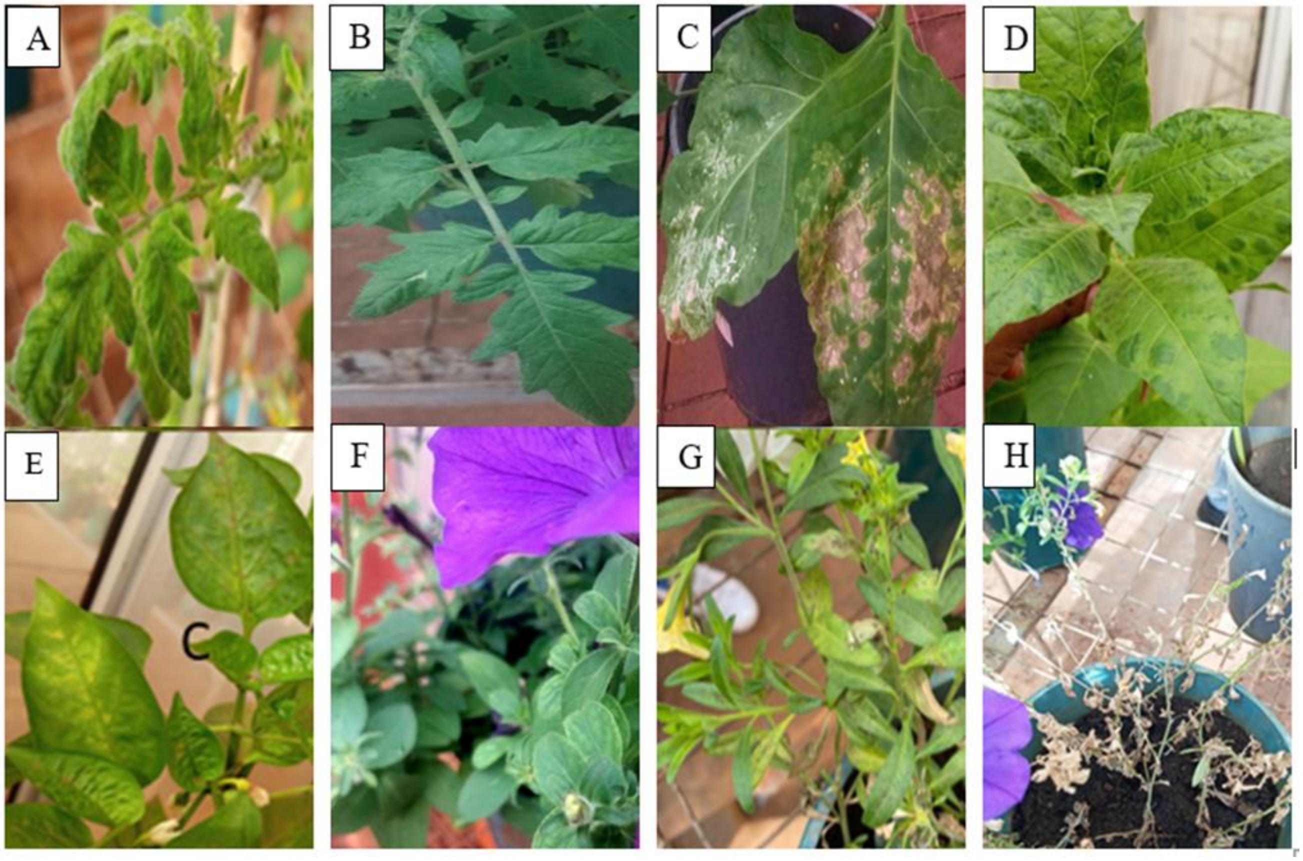

S2 Fig. Symptoms on selected indicator plants mechanically inoculated with ToMV isolates

A) *Solanum lycopersicum* (money maker) B) *Solanum lycopersicum* (Asilla F1) C & D *Nicotiana tabacum cv. Samsun*, E) *Capsicum annum* F) *Petunia x hybrida,* G& H) *Calibrachoa parviflora*
