# Supplementary material for: A reverse transcription loop-mediated isothermal amplification assay for quick detection of tomato mosaic virus
Source: PLoS One. 2024 Jun 13;19(6):e0304497. doi: 10.1371/journal.pone.0304497 (PMC11175515; doi:10.1371/journal.pone.0304497)
Supplement: S3 Fig — (PDF) [file pone.0304497.s003.pdf]

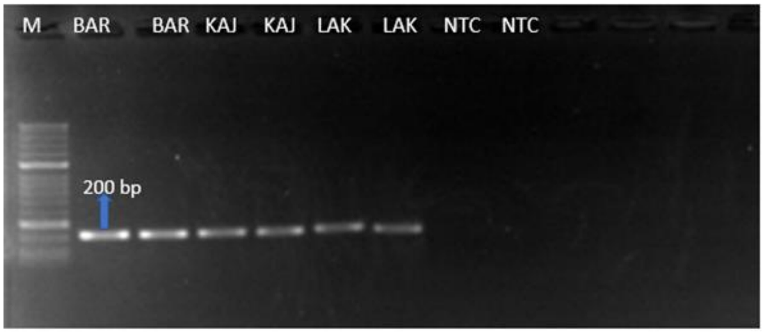

S3 Fig. Agarose gel electrophoresis of PCR amplified products (200 bp) of tomato mosaic virus coat protein gene using outer primers F3 and B3. Lane M-50 bp Hyper Ladder (Bioline) ; Lanes 2 to 7-ToMV isolates from three different counties in Kenya; Lanes 8 and 9- non-template control
