# Supplementary material for: A reverse transcription loop-mediated isothermal amplification assay for quick detection of tomato mosaic virus
Source: PLoS One. 2024 Jun 13;19(6):e0304497. doi: 10.1371/journal.pone.0304497 (PMC11175515; doi:10.1371/journal.pone.0304497)
Supplement: S4 Fig — (PDF) [file pone.0304497.s004.pdf]

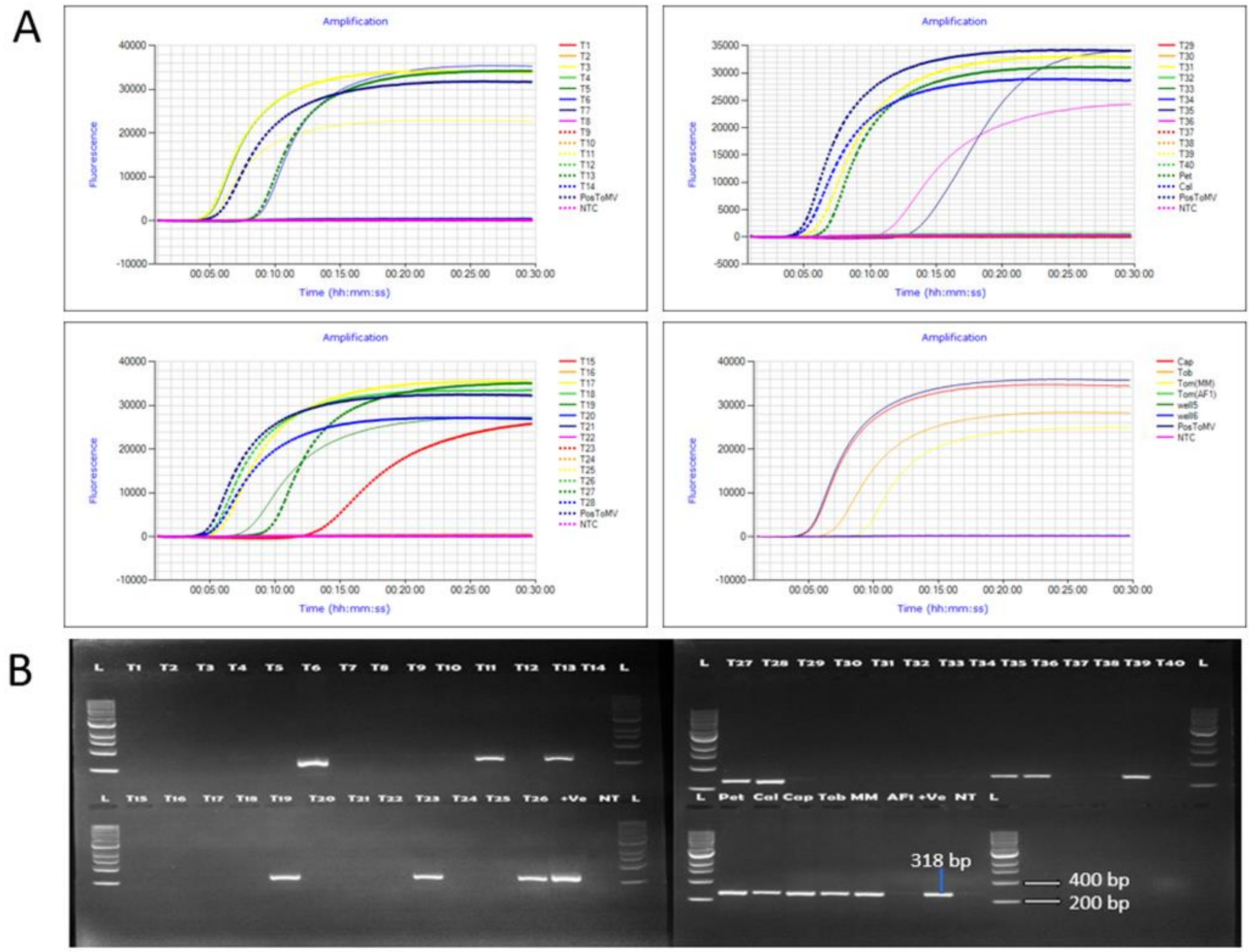

S4 Fig. Evaluation of ToMV RT-LAMP assay

(A) Fluorescence detection of LAMP products (B) Agarose gel electrophoresis analysis, Lane L: 1kb bp ladder (Bioline); T1 to T40: tomato leaf samples from the field; Pet-petunia; Cal-Calibrachoa; Cap-Capsicum; Tob-Tobacco, Tom (MM)-Tomato moneymaker; Tom (A. F1)-Tomato Asilla F1; NT-no template control; +positive control
