# Supplementary material for: A reverse transcription loop-mediated isothermal amplification assay for quick detection of tomato mosaic virus
Source: PLoS One. 2024 Jun 13;19(6):e0304497. doi: 10.1371/journal.pone.0304497 (PMC11175515; doi:10.1371/journal.pone.0304497)
Supplement: S1 Table — (DOCX) [file pone.0304497.s005.docx]

**S1 Table. Amplification time and melting temperatures of the four tomato samples used in optimization**

| **Sample code** | **RNA extraction method** | | | |
| --- | --- | --- | --- | --- |
|  | **CTAB** | | **APEG** | |
|  | **Time (mm:ss)** | **Anneal ℃** | **Time (mm:ss)** | **Anneal ℃** |
| BAR 1 | 5:30 | 85.84 | 6:30 | 87.45 |
| BAR 1 | 5:30 | 85.84 | 6:45 | 87.65 |
| BAR 2 | 6:30 | 85.90 | 5:00 | 85.60 |
| BAR 2 | 6:30 | 85.90 | 5:00 | 85.60 |
| KAJ 1 | 4:45 | 85.80 | 5:30 | 86.60 |
| KAJ 1 | 4:45 | 85.80 | 5:30 | 86.60 |
| LAK 3 | 5:15 | 85.82 | 6:30 | 88.70 |
| LAK 3 | 5:15 | 85.82 | 6:30 | 88.70 |
| HC | - | - | - | - |
| HC | - | - | - | - |
| NTC | - | - | - | - |
| NTC | - | - | - | - |
| POS | 6:15 | 85.89 | 9:30 | 87.50 |
| POS | 6:15 | 85.89 | 10:00 | 87.55 |
