# Supplementary material for: A reverse transcription loop-mediated isothermal amplification assay for quick detection of tomato mosaic virus
Source: PLoS One. 2024 Jun 13;19(6):e0304497. doi: 10.1371/journal.pone.0304497 (PMC11175515; doi:10.1371/journal.pone.0304497)
Supplement: S1 Raw images — (PDF) [file pone.0304497.s006.pdf]

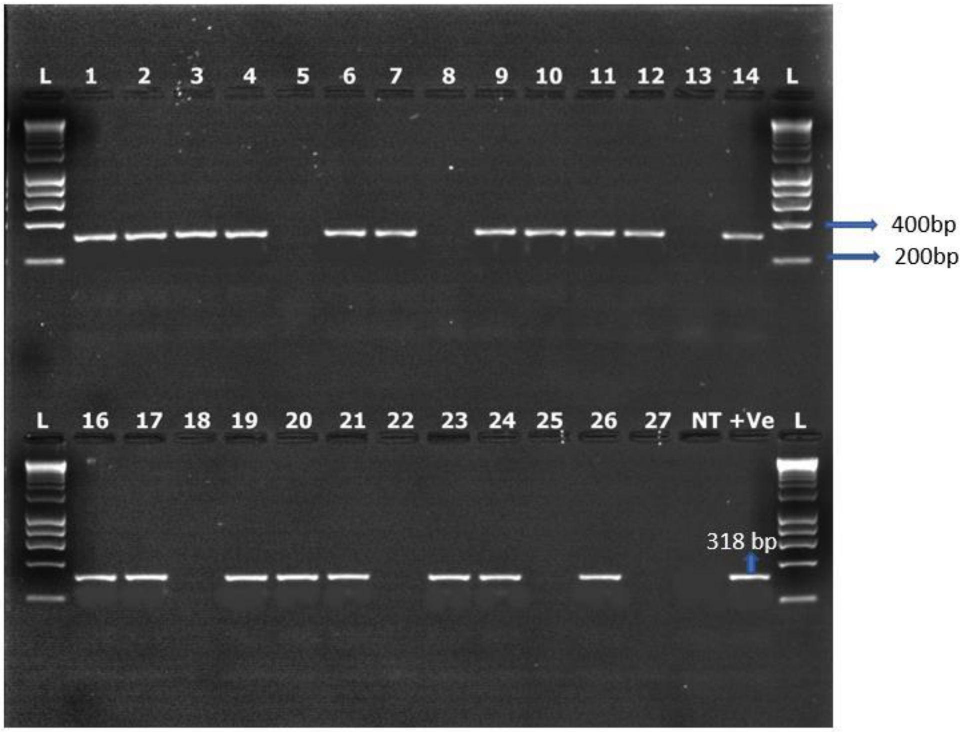

Detection of ToMV by RT-PCR.

L: 1Kb Hyper Ladder (Bioline); lane 1-27 samples; lane 28 –Non-template control; 29-Positive control

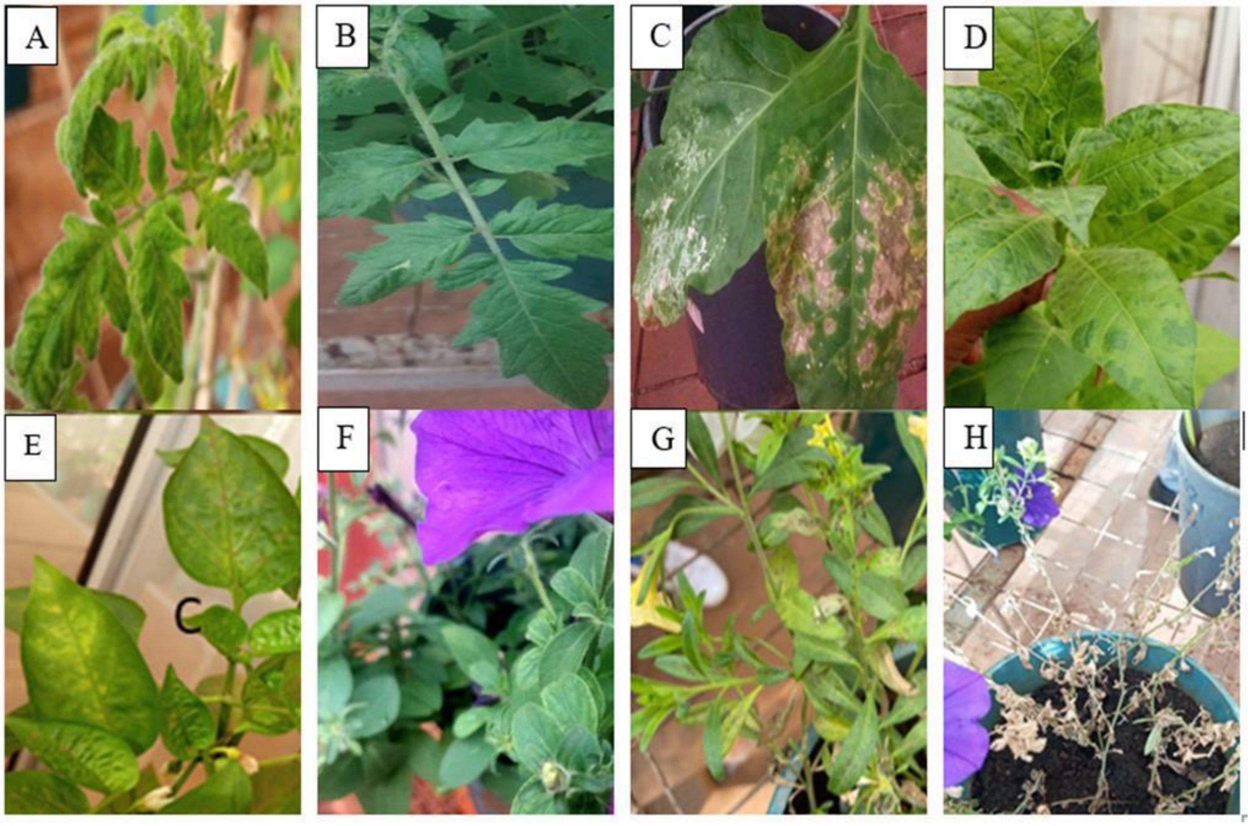

Symptoms on selected indicator plants mechanically inoculated with ToMV isolates.

A) *Solanum lycopersicum* (money maker) B) *Solanum lycopersicum* (Asilla F1) C & D *Nicotiana tabacum cv. Samsun,* E) *Capsicum annum* F) *Petunia x hybrida,* G& H) *Calibrachoa parviflora*

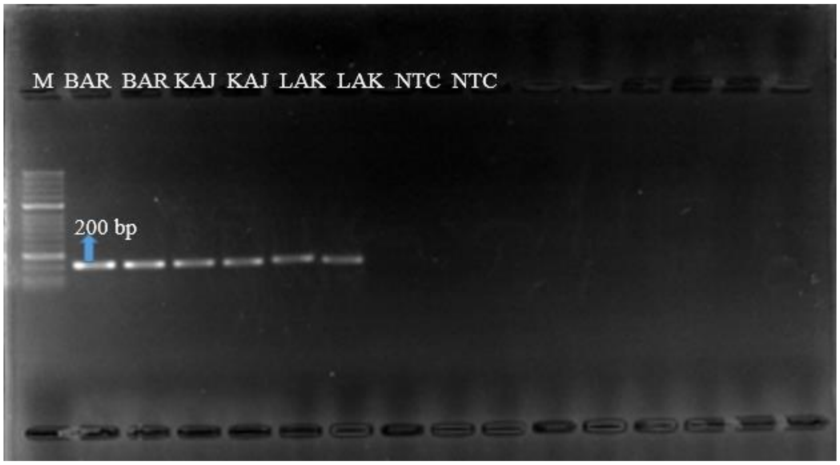

Agarose gel electrophoresis of PCR amplified products (200 bp) of tomato mosaic virus coat protein gene using outer primers F3 and B3.

Lane M-50 bp Hyper Ladder (Bioline) ; Lanes 2 to 7-ToMV isolates from three different counties in Kenya; Lanes 8 and 9- non-template control

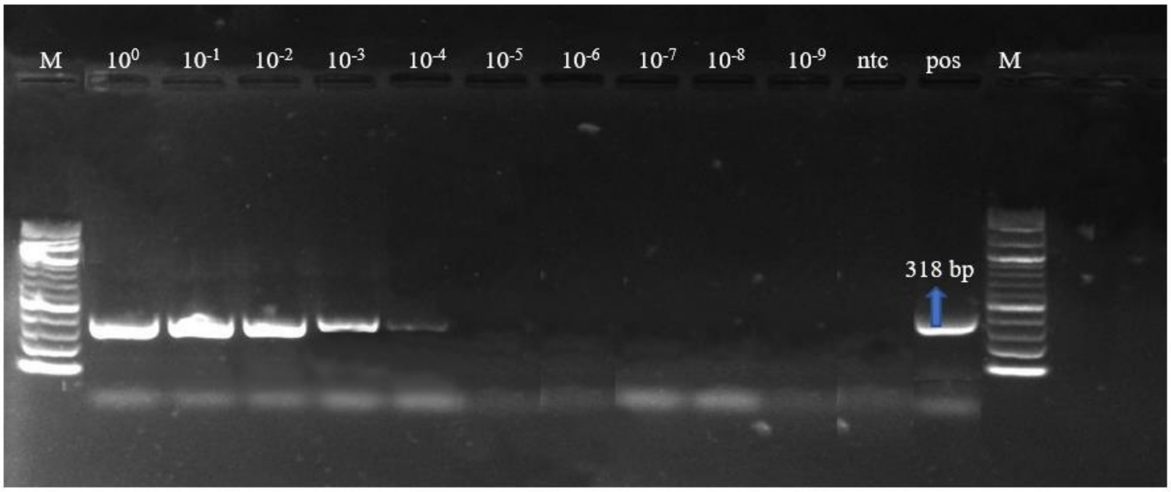

Fig 3C. Sensitivity determination of RT-PCR.

ToMV amplification by RT-PCR from purified total RNA; M-1Kb plus DNA ladder (New England Biolabs); lanes 2-11-RNA extracted using CTAB from ToMV infected plant and diluted in series ranging from 100 ng/µL to 10-9 ng/µL; ntc-non-template control; pos-positive control

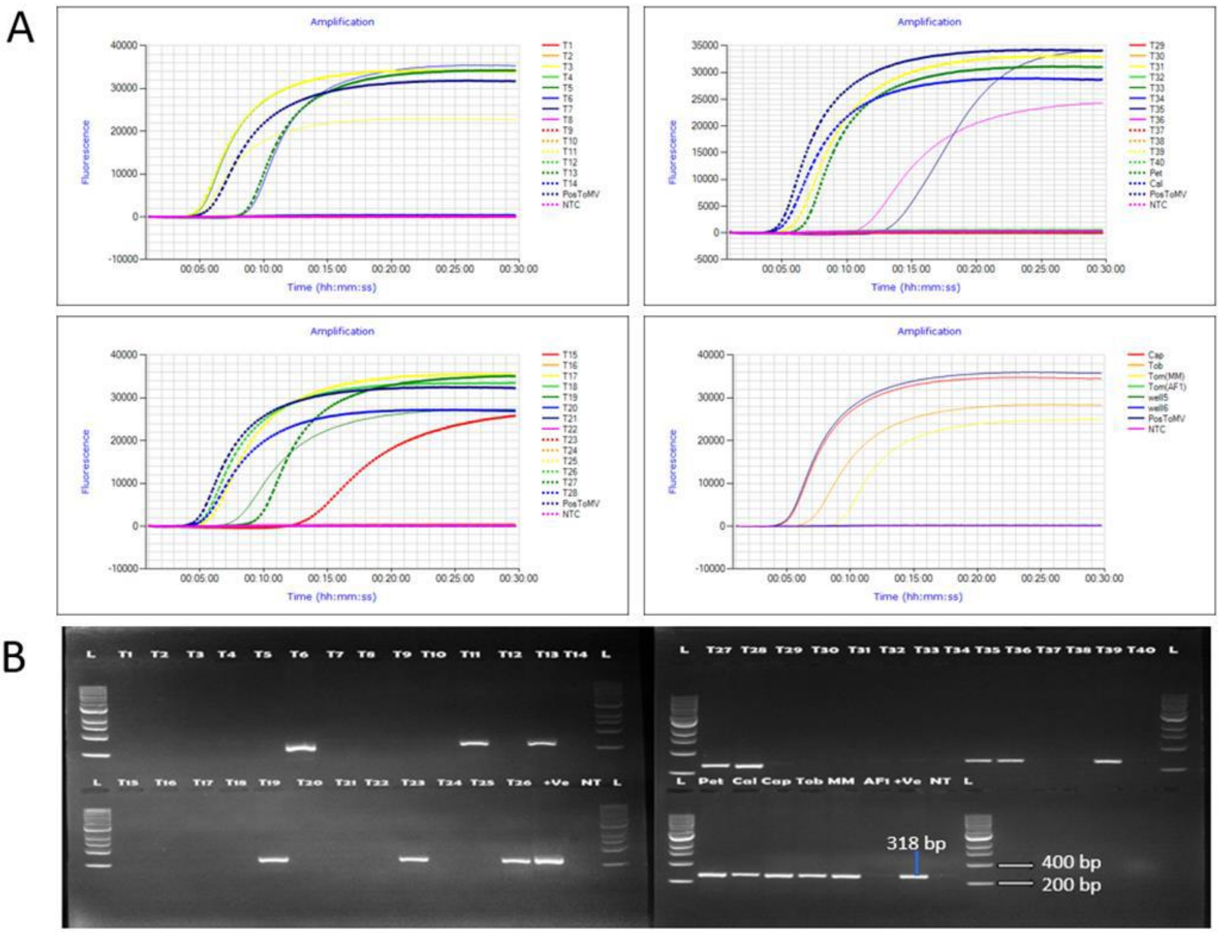

Evaluation of ToMV RT-LAMP assay

(A) Fluorescence detection of LAMP products (B) Agarose gel electrophoresis analysis, Lane L: 1kb bp ladder (Bioline); T1 to T40: tomato leaf samples from the field; Pet-petunia; Cal-Calibrachoa; Cap-Capsicum; Tob-Tobacco, Tom (MM)-Tomato moneymaker; Tom (A. F1)-Tomato Asilla F1; NT-no template control; +positive control
